# Micafungin concentrations 100 mg daily in plasma and burn eschars in patients with severe burn injuries

**DOI:** 10.1186/cc14192

**Published:** 2015-03-16

**Authors:** A Agrifoglio, MJ Asensio, M Sánchez, B Galván, E Herrero, L Cachafeiro, E Perales, S Luque, A García de Lorenzo

**Affiliations:** 1 La Paz/IdiPAZ University Hospital, Madrid, Spain; 2 Hospital del Mar, Barcelona, Spain

## Introduction

Micafungin (MCF) is an echinocandin agent with broad activity against *Candida *spp., which are frequently isolated in blood and eschar cultures of burned patients, who present different pharmacokinetics (PK) characteristics. Due to the limited information about its PK, we investigate MCF levels in plasma and burn eschar tissues in this population.

## Methods

A PK study of MCF at standard dosage (100 mg/day). Cmax (end of the infusion) and Cmin (before next dose) plasma levels of MCF were obtained after first dose and at steady state (days 4 and 5 of therapy); and on day 5 in eschars (1 to 3 hours after infusion). They were measured by HPLC. Spearman's rho test was used for bivariate correlations between MCF exposure and patient's clinical factors.

## Results

There were 10 patients (eight men; age: 18 to 77 years). Patients' characteristics and PK are shown in Table 1. A high interindividual variability was observed in the concentrations of MCF. Peak plasma concentrations after the first and repeated doses of MCF were inversely correlated with % burned TBSA (Spearman's ρ = -0.695 and -0.750 (*P *< 0.05)), respectively, but not with the time from burn injury. MCF concentrations in burn eschars were not correlated with % burned TBSA. MCF was well tolerated. One patient had candidemia. The crude mortality was 40%.

**Table 1:** Clinical and pharmacokinetic characteristics of patients.

Patient	% burned TBSA	% FT	ABSI	MCF dose (mg/kg body weight)	Plasma Cmax/Cmin after first dose (μg/ml)	Plasma C/max/Cmin at steady state (μg/ml)	Burn easchar tissue on day 5 (μg/ml)	Days from admission to the start of MCF	SOFA at the beginning of MCF	LOS in BUCU (days)
1	35	20	9	1.3	8.6/0.8	7.4/1.0	2.3	38	1	75
2	40	35	8	2.0	8.5/1.1	9.4/1.8	<LQ	15	6	23
3	23	16	8	1.3	6.4/0.8	10.3/1.2	<LQ	12	2	17
4	70	40	12	1.1	3.9/0.5	4.5/0.8	0.4	8	6	43
5	23	12	7	1.3	7.5/1.8	8.0/1.4	0.6	10	5	19
6	70	60	11	1.2	3.4/0.5	5.0/0.9	1.5	12	5	70
7	80	70	12	1.1	3.8/0.4	4.0/0.4	0.2	34	2	61
8	60	50	10	1.4	4.8/0.5	4.3/1.0	0.2	34	2	90
9	44	34	10	1.3	4.5/1.1	9.1/2.3	0.2	8	6	34
10	34	28	9	1.3	4.1/0.7	5.4/1.0	0.7	10	5	35
Median	42	34.5	9.5	1.3	4.7/0.7	6.4/1.0	0.5	12	5.0	39
IQR	31.3 to 70.0	19.0 to 52.5	8 to 11.3	1.1 to 1.4	3.9 to 7.5/0.5 to 1.1	4.5 to 9.1/0.9 to 1.4	0.3 to 1.1	9.5 to 19.8	3.5 to 5.6	22.7 to 71.3

ABSI, Abbreviated Burn Severity Index; BICU, burn intensive care unit; FT, full thickness; IQR, interquartile range; LOS, length of hospital stay; LQ, limit of quantification (<0.1 μg/ml); SOFA, Sequential Organ Failure Assessment; TBSA, total body surface area.

## Conclusion

This is the largest PK study of 100 mg daily of MCF in severely burned critically ill patients. The inverse correlation between MCF exposure and % burned TBSA suggests that patients with large burned TBSA may need higher doses of MCF. Nevertheless, MCF levels in plasma and burn eschar tissues after first and multiple doses were above the MIC90 against most clinically important *Candida *species.

